# Characterizing thrombus adhesion strength on common cardiovascular device materials

**DOI:** 10.3389/fbioe.2024.1438359

**Published:** 2024-08-14

**Authors:** Vikas Kannojiya, Sara E. Almasy, Jose L. Monclova, Jerry Contreras, Francesco Costanzo, Keefe B. Manning

**Affiliations:** ^1^ Department of Biomedical Engineering, The Pennsylvania State University, University Park, PA, United States; ^2^ Department of Engineering Science and Mechanics, The Pennsylvania State University, University Park, PA, United States; ^3^ Department of Surgery, Penn State College of Medicine, Penn State Hershey Medical Center, Hershey, PA, United States

**Keywords:** cardiovascular medical devices, blood clot, adhesion, embolization, polymers, nitinol, titanium, stress

## Abstract

Thrombus formation in blood-contacting medical devices is a major concern in the medical device industry, limiting the clinical efficacy of these devices. Further, a locally formed clot within the device has the potential to detach from the surface, posing a risk of embolization. Clot embolization from blood-contacting cardiovascular devices can result in serious complications like acute ischemic stroke and myocardial infarction. Therefore, clot embolization associated with device-induced thrombosis can be life-threatening and requires an enhanced fundamental understanding of embolization characteristics to come up with advanced intervention strategies. Therefore, this work aims to investigate the adhesive characteristics of blood clots on common biocompatible materials used in various cardiovascular devices. This study focuses on characterizing the adhesion strength of blood clots on materials such as polytetrafluoroethylene (PTFE), polyurethane (PU), polyether ether ketone (PEEK), nitinol, and titanium, frequently used in medical devices. In addition, the effect of incubation time on clot adhesion is explored. Results from this work demonstrated strongest clot adhesion to titanium with 3 h of incubation resulting in 1.06 ± 0.20 kPa detachment stresses. The clot adhesion strength on titanium was 51.5% higher than PEEK, 35.9% higher than PTFE, 63.1% higher than PU, and 35.4% higher than nitinol. Further, adhesion strength increases with incubation time for all materials. The percentage increase in detachment stress over incubation time (ranging from 30 min to 3 h) for polymers ranged from at least 108.75% (PEEK), 140.74% (PU), to 151.61% (PTFE). Whereas, for metallic surfaces, the percentage rise ranged from 70.21% (nitinol) to 89.28% (titanium). Confocal fluorescence imaging of clot remnants on the material surfaces revealed a well-bounded platelet-fibrin network at the residual region, representing a comparatively higher adhesive region than the non-residual zone of the surface.

## 1 Introduction

Blood-contacting medical devices like catheters, vascular grafts, heart valves, stents, extracorporeal membrane oxygenation, and mechanical circulatory support (MCS) devices are widely used to treat cardiovascular pathologies (Jaffer et al., 2015a). These devices are an integral part of advanced medical treatment and can be used in an acute setting or as destination therapy. There is an array of biomaterials used with these devices, including metals, ceramics, polymers, and composites (Ahmed et al., 2020). Materials like polytetrafluoroethylene (PTFE), polyurethane (PU), polyether ether ketone (PEEK), nitinol, and titanium are most common for these devices (Festas et al., 2020). For instance, titanium and its alloys are used in MCS devices, nitinol in stents, PEEK in heart valves, PTFE in heart valve rings, and PU in heart valves and catheters (Sin et al., 2009; Jaganathan et al., 2014; Tateishi et al., 2014; Alipour et al., 2022; Crago et al., 2022). These materials are selected based on their biocompatibility, mechanical properties, durability, toxicity, surface characteristics, and cost (Bullock and Bussy, 2019; Alipour et al., 2022). However, none of these materials are completely biocompatible. As a result, thrombus formation can occur on the surface of blood-contacting devices, limiting their clinical success (Sarkar et al., 2007; Dangas et al., 2016; Wall et al., 2016).

Regardless of device type and duration of use, the contact of a foreign surface with blood can trigger primary and secondary hemostasis, resulting in clot formation (Jaffer et al., 2015a). Research on the improvement of biocompatibility of blood-contacting materials has advanced significantly over time. The progress includes the development of novel coatings, alloys, and surface engineering (Manivasagam et al., 2021; Eren et al., 2022). Despite biocompatibility advancement, thrombus formation continues to occur on cardiovascular devices (Jaffer et al., 2015b; Kreuziger et al., 2015; Taylor et al., 2016; Kreuziger et al., 2017). For instance, MCS has frequent events of thrombus formation with a reported incidence of 5.5%–12.2% (Kirklin et al., 2014; Najjar et al., 2014; Starling et al., 2014). Thrombus events are also evident in clinical valves following the transcatheter aortic valve implantation (TAVI) procedure with an approximate incidence of 7.2% (Abdel-Wahab et al., 2018). Clotting is amongst the most feared complications in stents due to its higher associated mortality rate which ranges from 11% to 42% (Kumar et al., 2022). Furthermore, the problem of thrombus formation is also common in catheters (incidence: 14%–30%) (Lee and Kamphuisen, 2012; Hrdy et al., 2017; Ostlund et al., 2019). Rare events of device thrombosis also have been noticed for defibrillators and pacemakers (Adar et al., 2019; Sulemane et al., 2020). A locally formed clot in, or on, the device poses the risk of embolization, which may get lodged downstream in a smaller vessel. Due to arterial blockage, the possibility of acute ischemic stroke (AIS) and myocardial infarction may increase. In this regard, Cho et al., 2019 observed that thrombosis in a MCS device could result in higher stroke events (Cho et al., 2019) noting that device thrombosis accounts for 31% of the total stroke patients treated with MCS. Similarly, reports show higher myocardial infarction events due to coronary stent thrombosis (D'Ascenzo et al., 2013). Therefore, clot embolization associated with device-induced thrombosis can be life-threatening and requires an enhanced fundamental understanding of embolization characteristics to come up with advanced surface modification and intervention strategies targeting improved hemocompatible surfaces.

As per Virchow’s triad, device-induced thrombosis is often attributed to interactions between blood, flow, and surfaces (Manning et al., 2021). Blood state, which involves factors such as activated platelets, patient-specific pathophysiology, and active coagulation due to vessel injury has a substantial impact on medical device clotting (Deutsch et al., 2006). Researchers have shown that flow-related features like recirculation, low and high shear stress, and shear rate can influence device-induced thrombosis (Balasubramanian and Slack, 2001; Navitsky et al., 2013). Similarly, the interaction of blood and foreign surfaces play a significant role in medical device clotting. Blood and foreign surface interaction vary from surface to surface (Xu et al., 2019; Vlcek et al., 2021) and may form clots of different characteristics. In particular, the adhesion between the blood clot and the biomaterial surface may vary. Accordingly, a different embolization behavior of the blood clot may be observed in such devices, which may be a material-dependent behavior of clot adhesion. Studies have described the micromechanical interaction and adhesion of blood cells and platelets on different materials (Pierrat et al., 2004; Koh et al., 2010; Cai et al., 2020). However, the understanding of adhesion strength of macroscopic blood clots is lacking. Therefore, this study aims to characterize the adhesive strength of blood clots formed on common cardiovascular biomaterials to aid in understanding the device-associated embolic complications like AIS and myocardial infarction.

## 2 Materials and methods

### 2.1 Material characterization

Among commonly used materials in cardiovascular medical devices, the following five materials were used in this study: polytetrafluoroethylene (PTFE), polyurethane (PU), polyether ether ketone (PEEK), Nitinol, and Titanium (Ti-6Al-4V). All these materials were commercially available (McMaster Carr, United States of America and Kellog’s Research Lab, United States of America), and samples were cut into 25 mm square pieces. All samples were cleaned with ethanol to remove any residue from the material cutting, and subsequently, dried before testing. Six samples from each material were tested using blood from six individual donors at three distinct incubation periods. Optical profilometry (Zeiss LSM880, Zeiss, United States of America) was also used to characterize the surface. The average roughness (Sq) values of different materials were summarized in Table 1. The roughness data show the surface was reasonably flat, further the roughness of these materials was consistent with previous descriptions (Clarke et al., 2007; Linneweber et al., 2007; Mi et al., 2018; Segan et al., 2020; Chen et al., 2023; Jayaraman et al., 2023).

**TABLE 1:** Average roughness value of biocompatible materials at ×50 confocal objective and ×2 field of view.

	Nitinol	PEEK	Polyurethane	PTFE	Titanium
Sq (μm)	0.48 ± 0.04	0.01 ± 0.00	0.25 ± 0.03	0.20 ± 0.04	0.51 ± 0.13

### 2.2 Blood clot preparation

To study the effect of adhesion, clots were created using human blood, following an approved IRB protocol. Briefly, human whole blood was anticoagulated with 0.32% wt. sodium citrate (ThermoFisher, Waltham, MA, United States of America). Blood was separated into red blood cells, platelet-rich plasma (PRP), and platelet-poor plasma (PPP) *via* centrifugation and then reconstituted to 214 × 10^6^ platelets per mL, and hematocrit was controlled by the addition of 40% vol. red blood cells. To counter the effects of the anticoagulant, blood was recalcified with 20 mM calcium chloride (CaCl_2,_ Millipore Sigma, Burlington, MA, United States of America) and 0.25 NIH Unit/mL thrombin from human plasma (BioPharm, Bluffdale, UT, United States of America) to initiate coagulation. The blood was then immediately injected into resin-based (Clear V4, Formlabs, Somerville, MA, United States of America) cylindrical molds (14 mm in diameter and 10 mm high) attached to the different materials. The injected blood was incubated to allow clot formation over durations of 30 min, 1 h, and 3 h at 37°C, prior to testing.

### 2.3 Mechanical testing

**FIGURE 1:**
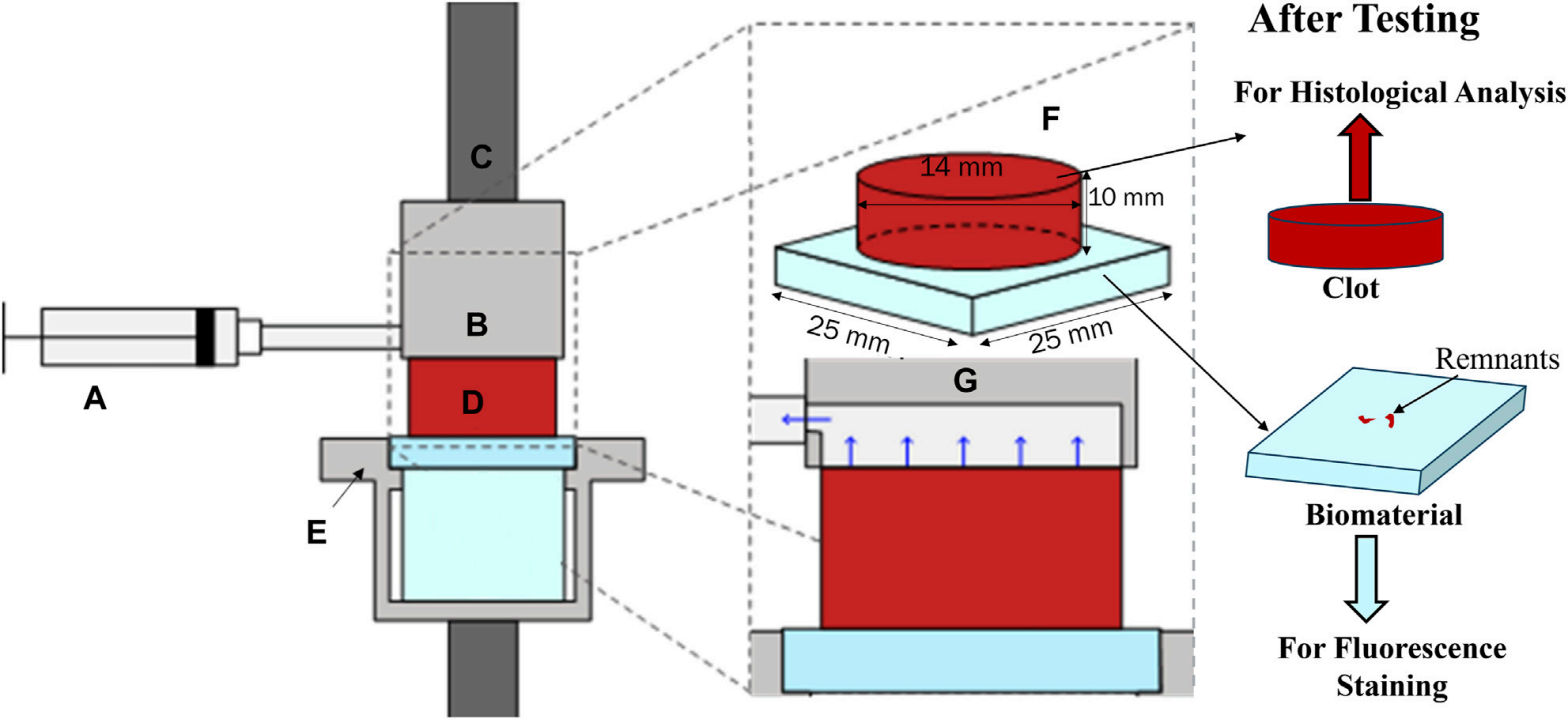
Adhesion test rig with **(A)** syringe applying suction through **(B)** a custom suction chamber fixed to **(C)** the top grip. **(D)**. The cylindrical clot specimen was fixed to a bottom fixture, **(E)**. **(F)**. Shows an isometric projection of the clot attached to the biomaterial, and **(G)**. shows a zoomed-in view of the suction mechanism. After testing, the clot samples were fixed in 4% paraformaldehyde (PFA) for histological analysis and biocompatible material samples were stained for fluorescent confocal microscopy.

Clot adhesion strength was characterized using a uniaxial load frame (Instron, Norwood, MA, United States of America) with a 50 N load cell. Clots adhered to the material surface were placed on the bottom platform of a custom detachment rig. The custom rig held the surface samples intact throughout the detachment experiments, as shown in Figure 1E. A suction fixture (Figure 1B) was attached to the load frame to grip the clot. The suction fixture moves in tandem with the load frame, applying a negative (suction) pressure of 200 mmHg to the top surface of the clot. This pressure was used to grip the clot with the assistance of a custom syringe fixture calibrated with a vacuum pump (Figure 1C). Once the suction was applied, the specimen was loaded at a rate of 10% strain per second until complete detachment from the bottom platform. From these data, a linear polynomial was fit to the nominal stress-strain curve prior to fracture to compute the modulus. The nominal peak stress and strain were recorded.

### 2.4 Fluorescence staining for clot components on the material surface

Referring to Figure 1, after mechanical testing, biocompatible material samples were stained for fluorescent confocal microscopy. Initially, the samples were rinsed with 1x PBS and then fixed with 1% PFA for 1 h at 4°C in the dark. Following this, the samples were rinsed again with PBS and permeabilized with 0.5% Triton X-100 (Sigma, St. Louis, MO, United States of America) in 1 mL of blocking agent (goat serum, Sigma, #G9023) in the dark for 1 h at room temperature. After another PBS rinse, the samples were incubated overnight at 4°C with 3 µL of platelet antibody (monoclonal anti-CD41, Sigma, Invitrogen Catalog #14-0419-82) in 1 mL of 5% goat serum. The samples were then rinsed with PBS and treated with 1.5 µL of secondary antibody (goat anti-mouse IgG, Alexa Fluor 488, Invitrogen Catalog #A-11001, ThermoFisher, Waltham, MA, United States of America) in 1 mL of goat serum in the dark at room temperature. Similarly, for fibrin detection, the samples were stained using 5 µL of primary fibrinogen antibody (anti-fibrinogen, gamma antibody, Invitrogen, Catalog #MA5-34761, ThermoFisher) and 2 µL of secondary fibrinogen antibody (Alexa Fluor™ 647, Catalog #F35200, ThermoFisher). Finally, the samples were rinsed with PBS and mounted with coverslips using ProLong™ Diamond Antifade Mountant with DAPI (Catalog #P36962, ThermoFisher). After staining, the samples were imaged using an Olympus IX71 inverted fluorescent microscope (Leica Microsystems, Germany). The raw images were then processed in Fiji (NIH, United States of America). A custom script was used to quantify the percentage of the image area covered by platelets and fibrin for each sample from the six donated blood samples. The mean and standard deviation of the data were calculated using Microsoft Excel (Redmond, WA, United States of America).

### 2.5 Histology

**FIGURE 2:**
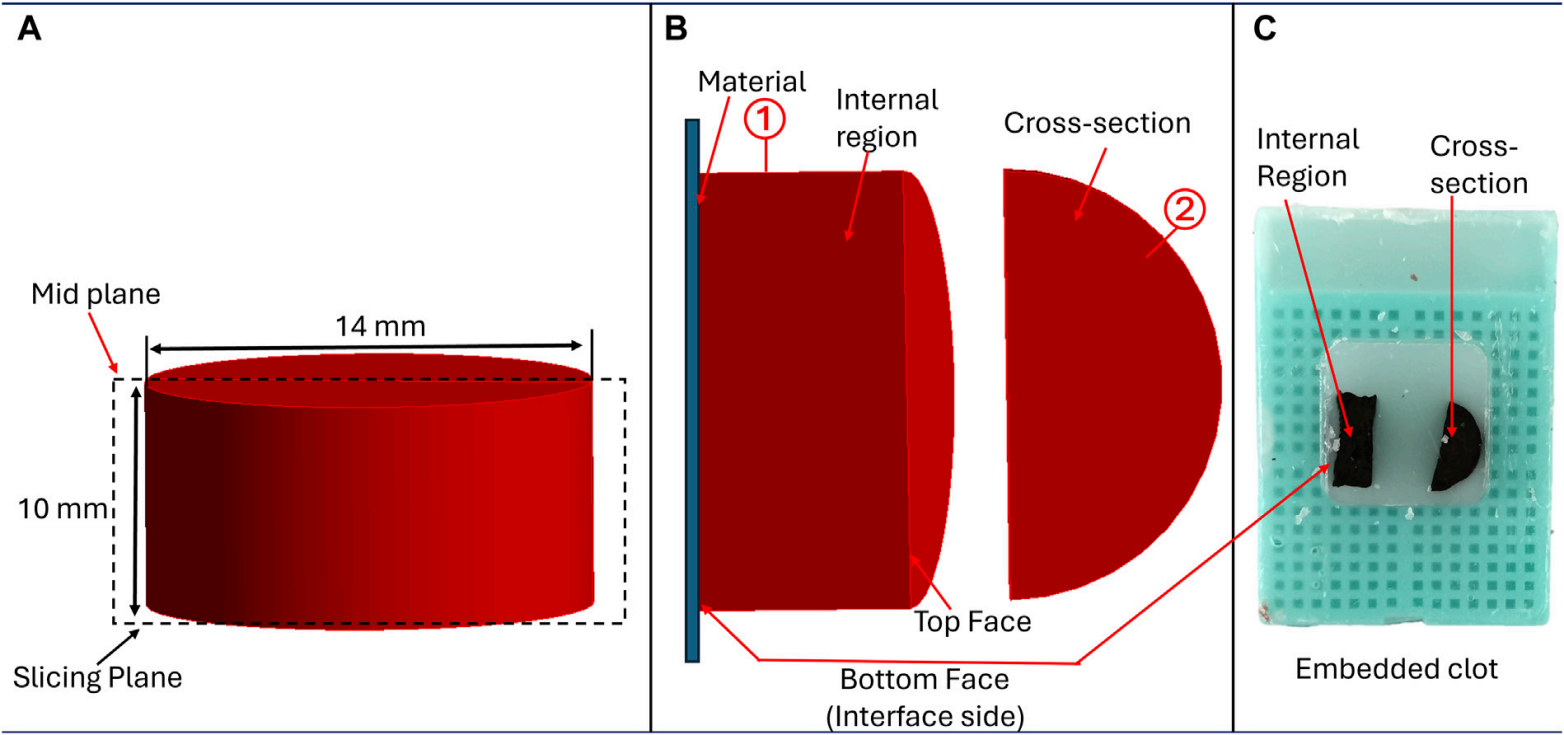
Methodology for sample preparation for histological analysis, **(A)** Cutting plane illustrating clot sectioning, **(B)** Positioning of clot samples to examine structural details at different regions, and **(C)** Clot samples embedded in paraffin for analysis.

Referring to Figure 1, after mechanical testing, clot samples were immediately fixed in 4% paraformaldehyde (PFA) for 48 h at 4°C for histological analysis. Clots were then cut into two halves by a vertical slicing plane (Figure 2A). The samples were then placed in the tissue cassettes such that the first half (labeled as ①, in Figure 2B) represents the cross-section of the clot, whereas the internal region of the clot is represented by the other half (labeled as ②). The top and bottom edges of the second half of the clot (rectangular part) also represent the edges of the top and bottom face (clot-material interface) of the clot, respectively (Figure 2B). All specimens were dehydrated in ethanol, followed by embedding in paraffin wax and sectioning into 6 µm slices for subsequent staining. A Carstairs protocol was used to stain the samples, facilitating visualization of the clot composition in terms of fibrin, platelets, and red blood cells. After staining, the samples were imaged using a fluorescent microscope (Olympus, Tokyo, Japan). The composition of platelet, fibrin, and red blood cells in terms of percentage was quantified for each clot sample by processing the histological images using a custom MATLAB script (v.2022b, MathWorks, Natick, MA, United States of America), wherein hue-saturation-value (HSV) threshold values were set to distinguish fibrin [red, hue = (0–10, 235–255)], platelets [navy blue, hue = (128–230)], and red blood cells [yellow, hue = (12–50)]. Subsequently, the mean and standard deviation of these percentage compositions were calculated to evaluate the average composition and variability within each blood sample. The variation in mean composition values across donors was characterized as the distribution.

### 2.6 Statistics

Blood samples from six individual donors (n = 6) were collected to form the clots. These clots were incubated for different times to study the effect of incubation time on adhesion strength. Three incubation times were used: 30 min, 1 h, and 3 h. For each incubation time, six clot replicates (n = 6) were formed from each donor’s blood. In total, 540 clot samples were formed and used in this study. Mean values, with corresponding standard deviation, were computed for each donor and across the donors. A two-way analysis of variance (ANOVA) was employed to compare the means across different material interfaces and incubation periods. All statistical analysis was completed using Microsoft Excel (Redmond, WA, United States of America). The statistical differences were indicated using asterisks (*) based on the *p*-values, where a single asterisk (*) represents a *p*-value of less than 0.05, two asterisks (**) represent a *p*-value of less than 0.01, and three asterisks (***) represent a *p*-value of less than 0.001 (Chad et al., 2018).

## 3 Results

Blood samples were incubated to form clots on various material surfaces at 30-min, 1-h, and 3-h intervals to investigate adhesion between blood clots and biocompatible surfaces. Multiple experiments were conducted to gather data on clot detachment from the surface, and the subsequent sections provide a comprehensive overview of the findings.

### 3.1 Detachment stress

**FIGURE 3:**
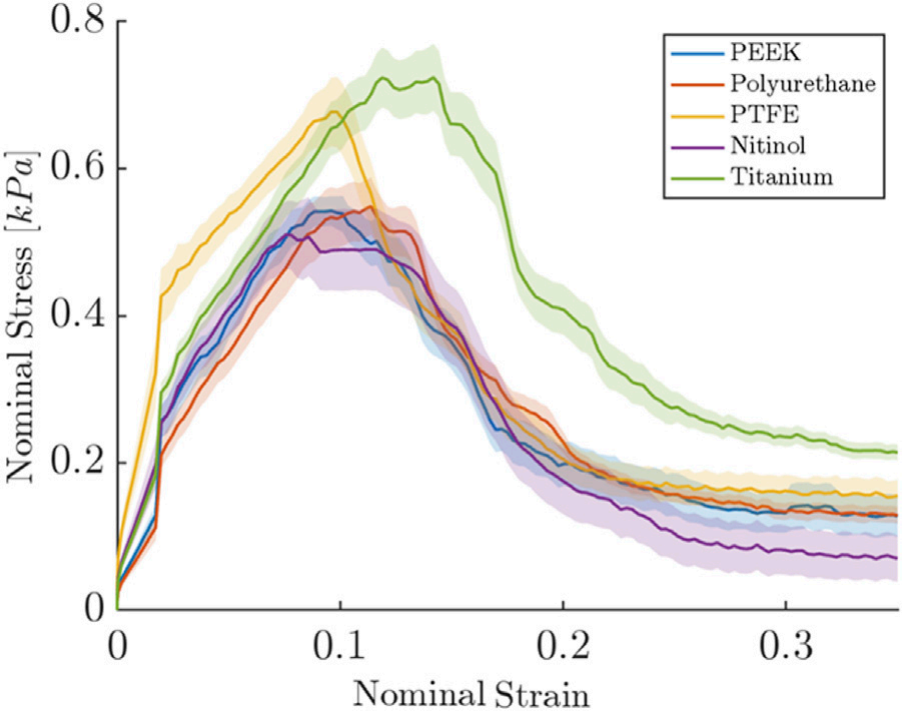
Nominal stress and strain relationships for clot during detachment from different surfaces. The results shown are for the samples incubated for 3 h. For each biomaterial, the solid line represents the nominal stress-strain response averaged over six repetitions of the test, with each test consisting of six replicate measurements. The shaded region represents the standard error.

The clot adhesion strength was quantified by measuring the nominal stress required to detach the clot from the material surface. Figure 3 depicted the variation of the nominal stress and strain in clots during the detachment from different surfaces. As the stress on the clot increases, the clot begins to deform until it reaches its peak stress, at which time the clot detaches from the surface (thus termed ‘detachment stress’), leading to a significant reduction in stress on the clot. Figure 3 represents the average detachment behavior of clots on each material, while Table 2 summarizes the actual mean nominal detachment (peak) stress values for different materials.

**TABLE 2:** Mean nominal detachment stress for different clot incubation times.

Material	Detachment stress (kPa)			Increase in adhesion strength
	30 min	1 h	3 h	From 30 min to 3 h of incubation (%)
PTFE	0.31 ± 0.05	0.50 ± 0.10	0.78 ± 0.20	151.61
Nitinol	0.47 ± 0.11	0.65 ± 0.19	0.77 ± 0.24	64.85
PU	0.27 ± 0.09	0.44 ± 0.06	0.65 ± 0.25	140.74
Titanium	0.56 ± 0.16	0.46 ± 0.06	1.06 ± 0.20	89.28
PEEK	0.32 ± 0.06	0.47 ± 0.8	0.70 ± 0.14	108.75

**FIGURE 4:**
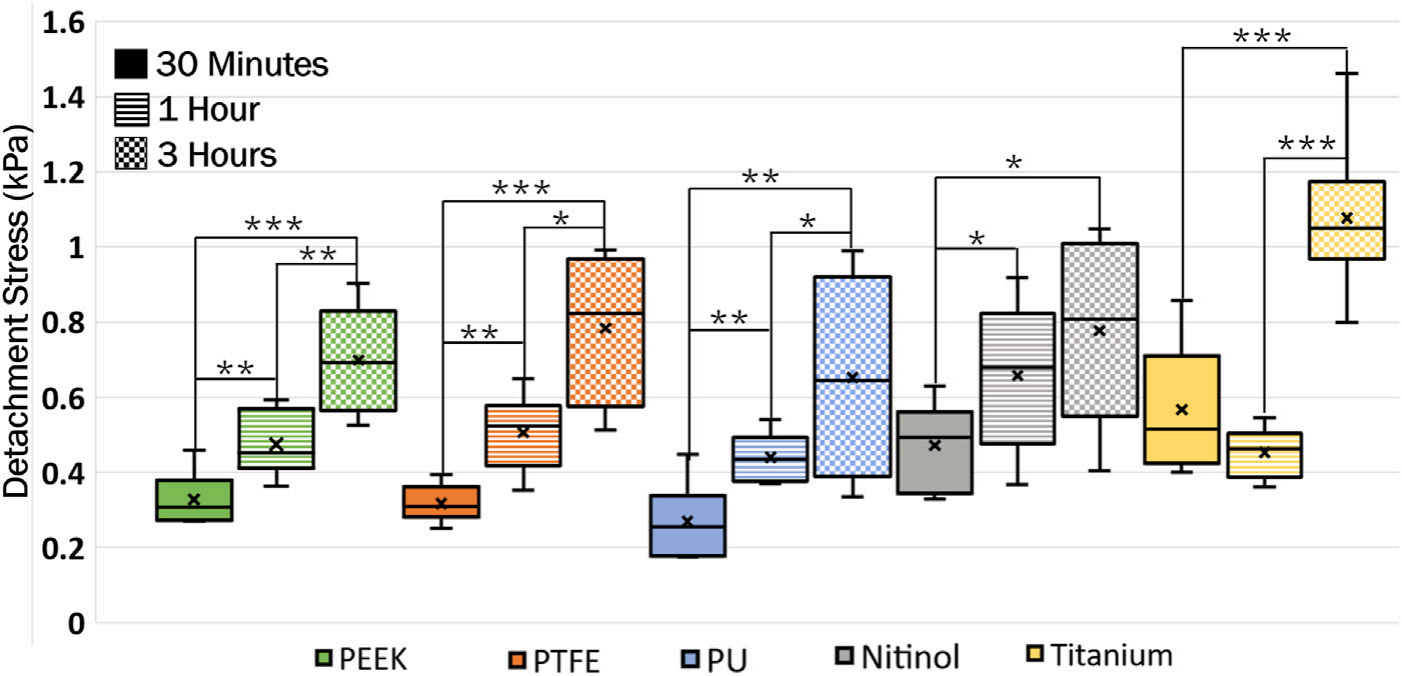
Data showing the distribution of detachment stress for clots attached to different surfaces at different incubation times. A time-dependent variation in clot adhesion strength was observed. The statistical difference is measured in terms of *p*-value; a lower *p*-value signifies higher significance. The level of significance difference is represented by an asterisk (*) on the plot, where *p* < 0.05 = *, *p* < 0.01 = **, and *p* < 0.001 = ***.

**FIGURE 5:**
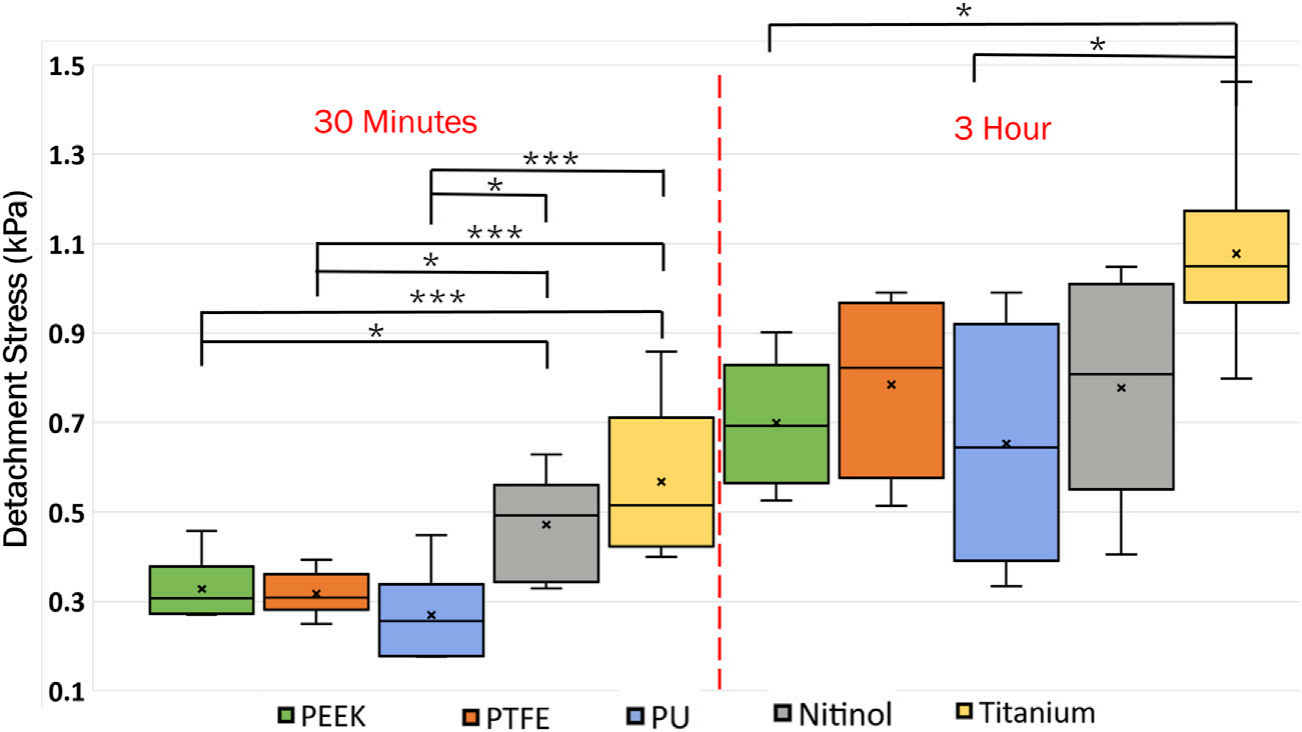
Distribution of clot detachment stress across different surface types during varied incubation periods. The adhesion strength of the clots showed variability based on the material type. That statistical difference is measured in terms of *p*-value; a lower *p*-value signifies higher significance. The level of significance difference is represented by an asterisk (*) on the plot, where *p* < 0.05 = *, and *p* < 0.001 = ***.

A higher detachment stress represents the stronger adhesion between the clot and the surface. Figure 4 depicted the detachment stress magnitude for clots incubated for 30 min, 1 h, and 3 h. The data showed that the detachment stress increases with incubation time, signifying that adhesion of blood clots had a time-dependent response on all surfaces. Overall, a positive correlation between incubation time and detachment stress was observed for all materials. Furthermore, a significant difference in clot adhesion strength was observed across different biomaterials at a given incubation time (Figure 5). Amongst all materials, clots on titanium showed the highest adhesion strength. On average, clots incubated on titanium surfaces for 3 h require 1.06 ± 0.20 kPa stress to be detached. The adhesion strength of clots on the titanium surface was approximately 35.89% higher than PTFE, 37.66% higher than nitinol, 51.48% higher than PEEK, and 63.06% higher than PU surface. The percentage increase of the detachment stress with time was observed to be higher in polymers than in metals (Table 2), suggesting greater influence of incubation time on clot adhesion for these polymer surfaces.

### 3.2 Clot remnants

**FIGURE 6:**
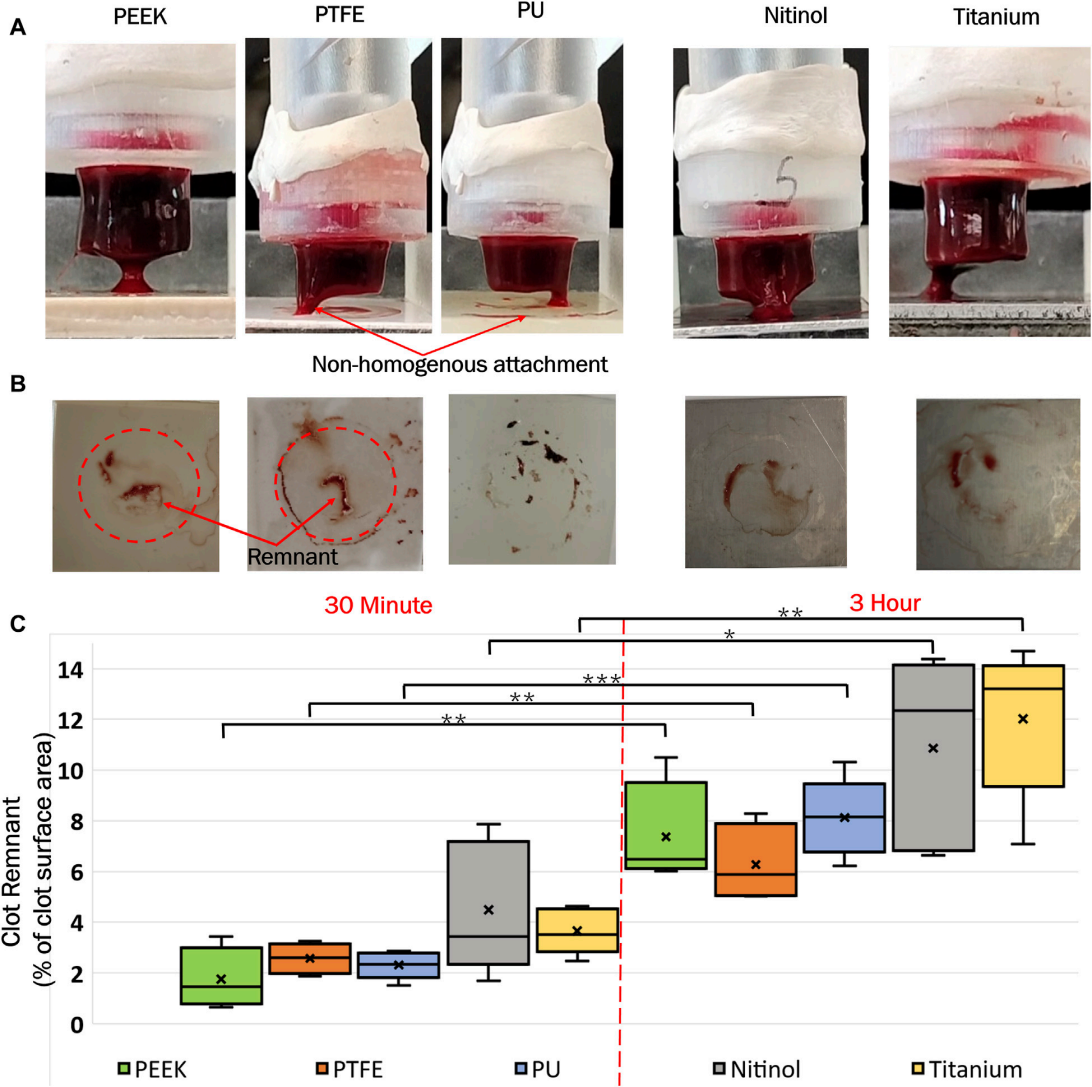
**(A)** Representative images of clot detachment on different surfaces, **(B)** Clot remnants on the surfaces after detachment, and **(C)** Distribution of clot remnants on materials at different incubation times. The level of significance difference is represented by an asterisk (*) on the plot, where *p* < 0.05 = *, *p* < 0.01 = **, and *p* < 0.001 = ***.

In the process of clot detachment, parts of the adhered clot exhibited prolonged adherence to biomaterials compared to other regions at the clot-biomaterial interface (representative images shown in Figure 6A). This irregular detachment represents nonhomogeneous attachment at the interface and some residual clot remnants remained on the surface post-detachment. Illustrative depictions of these remnants on different surfaces are shown in Figure 6B. A quantitative assessment of the remnants, based on surface area, was conducted using ImageJ software (National Institute of Health, Bethesda, MD, United States of America). The distribution of remnants in terms of percentage of clot surface area (area before the detachment) is presented in Figure 6C. The regions of clot remnants increased with incubation time on all surfaces. The remnant area represented the region of higher adhesive bonding of clot on the surface, which showed higher resistance the detachment. This illustrated that the greater adhesive regions also increased with the incubation time, with, more such regions observed on metallic surfaces (titanium and nitinol) compared to polymers.

**FIGURE 7:**
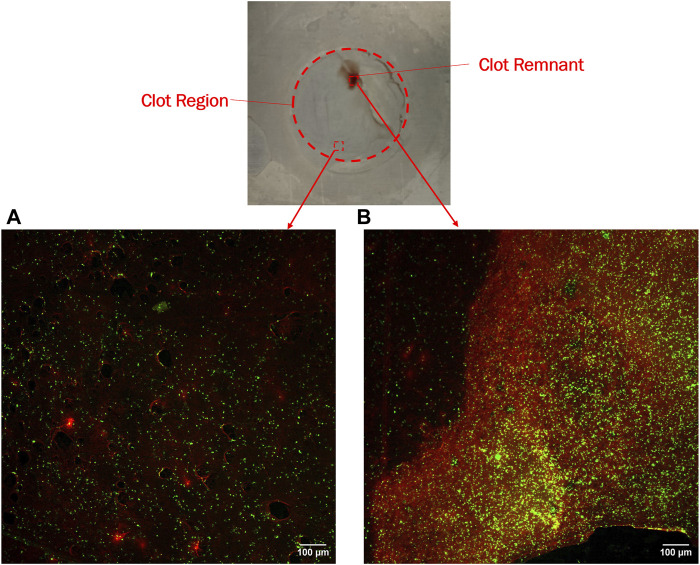
Confocal imaging of nitinol surface showing **(A)** Dispersed fibrin-platelet structure and **(B)** Bounded fibrin-platelet structure. Red and green colors represent fibrin and platelets, respectively. An Dispersed fibrin-platelet structure was noted in areas lacking residual clots. In contrast, regions containing residual clot remnants showed well-bounded platelet-fibrin distribution.

**FIGURE 8:**
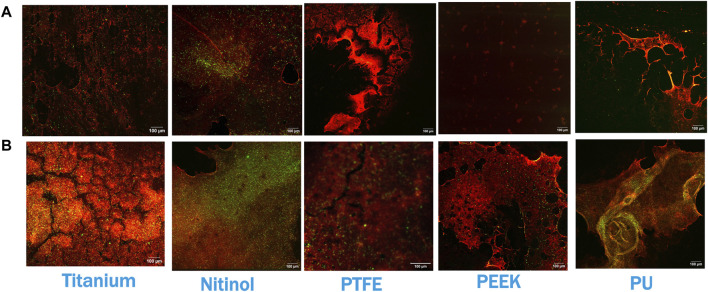
Confocal imaging at the residual region of different surfaces at **(A)** 30 min of incubation and **(B)** 3 h of incubation. Red and green colors represent fibrin and platelets, respectively. Images were captured at regions of the surface where clot remnants were present.

Confocal microscopy analysis was performed for all the materials to gain more insight into clot adhesion. Figure 7 illustrates the confocal image acquired from a nitinol surface at two distinct locations. An unbounded fibrin-platelet structure was observed on the surface where no clot residual was present, fewer platelets were seen in this region. However, in the region where clot residual remnants were present, a denser platelet region was observed. A well-bounded platelet-fibrin mesh could be observed in this region on all the materials, contributing to higher adhesion strength between clot and material. Due to the non-homogeneous distribution of the fibrin-platelet network, a non-uniform attachment of clots was seen at the interface of all the materials (Figure 6A). Figure 8 shows representative confocal images on the remnant region of different surfaces incubated for 30 min and 3 h. A denser fibrin mesh was observed at a higher incubation period on all materials (Supplementary Figure S2; Supplementary Material). Further, the region of well-connected platelet-fibrin mesh varies with the material type and increases further with time. This variation suggested that surfaces interact differently with platelets and fibrin, impacting the adhesion strength of the clots.

**FIGURE 9:**
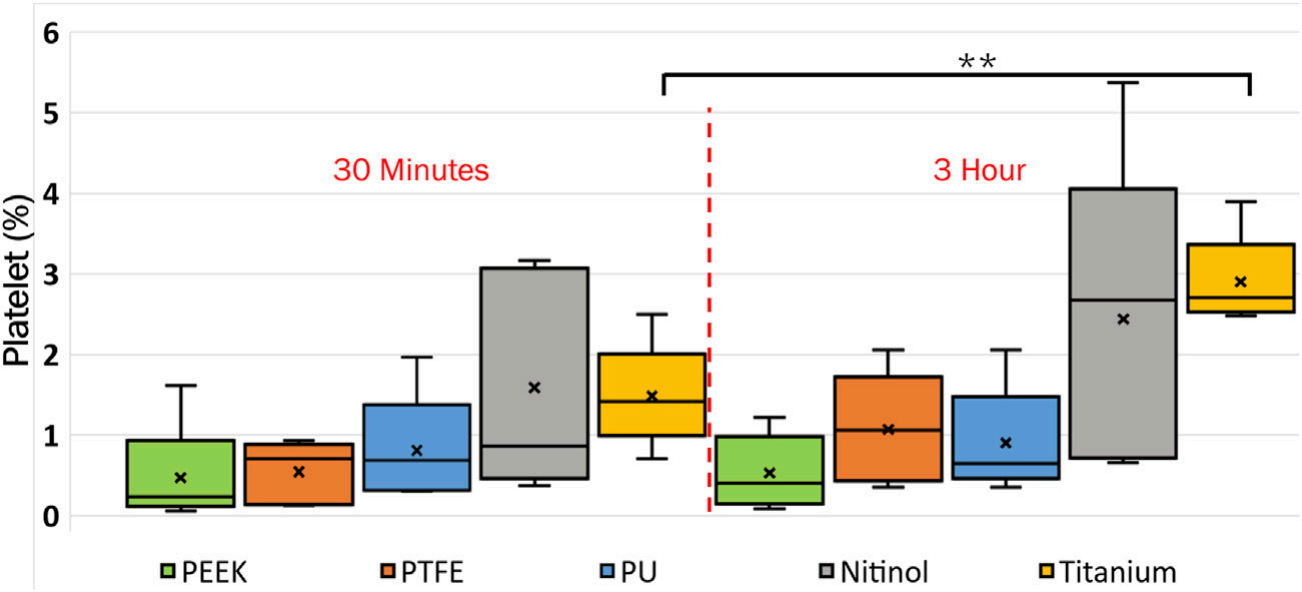
Quantification of platelets coverage on the biomaterial surfaces at different incubation times. After testing, the material samples were stained for fluorescent microscopy analysis and imaged using an Olympus IX71 inverted fluorescent microscope (Leica Microsystems, Germany). The raw images were then processed in Fiji (NIH, United States of America) to quantify the percentage of the image area covered by platelets using a custom script. Asterisks denote statistical significance between two-time intervals (*p* < 0.01 = **).

**FIGURE 10:**
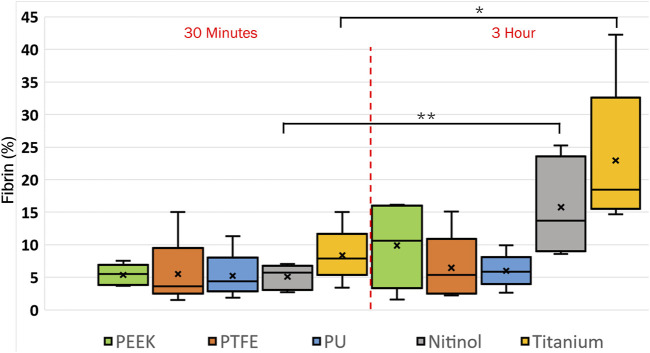
Quantification of fibrin coverage on the biomaterial surfaces at different incubation times obtained after quantifying the fluorescent microscopic images in Fiji. Asterisks denote statistical significance between two-time intervals (*p* < 0.05 = *, and *p* < 0.01 = **).

The platelet and fibrin distribution on the surfaces was quantified by taking an average of five images captured at fixed distinct locations within the clot region (top-left, top-right, bottom-left, bottom-right, and center) on the surface, excluding the remnant regions, on different surfaces at 30 min and 3 h of incubation. A considerable rise in platelet and fibrin distribution can be observed with time on each surface, Figures 9, 10. This indicates the temporal characteristics of surface-blood interactions, which exert a substantial influence on the adhesive behavior of blood clots. The temporal influence of blood-surface interactions was more evident on titanium and nitinol surfaces than on other materials. The percentage increase of platelet and fibrin with incubation time on the titanium surface was 95.42% and 173.66%, respectively. Whereas, the increment for the nitinol surface was about 53.77% and 210.55%, for the platelets and fibrin, respectively. The quantification of the platelet and fibrin over the remnant region is provided in (Supplementary Figures S1, S2).

### 3.3 Clot histological analysis

**FIGURE 11:**
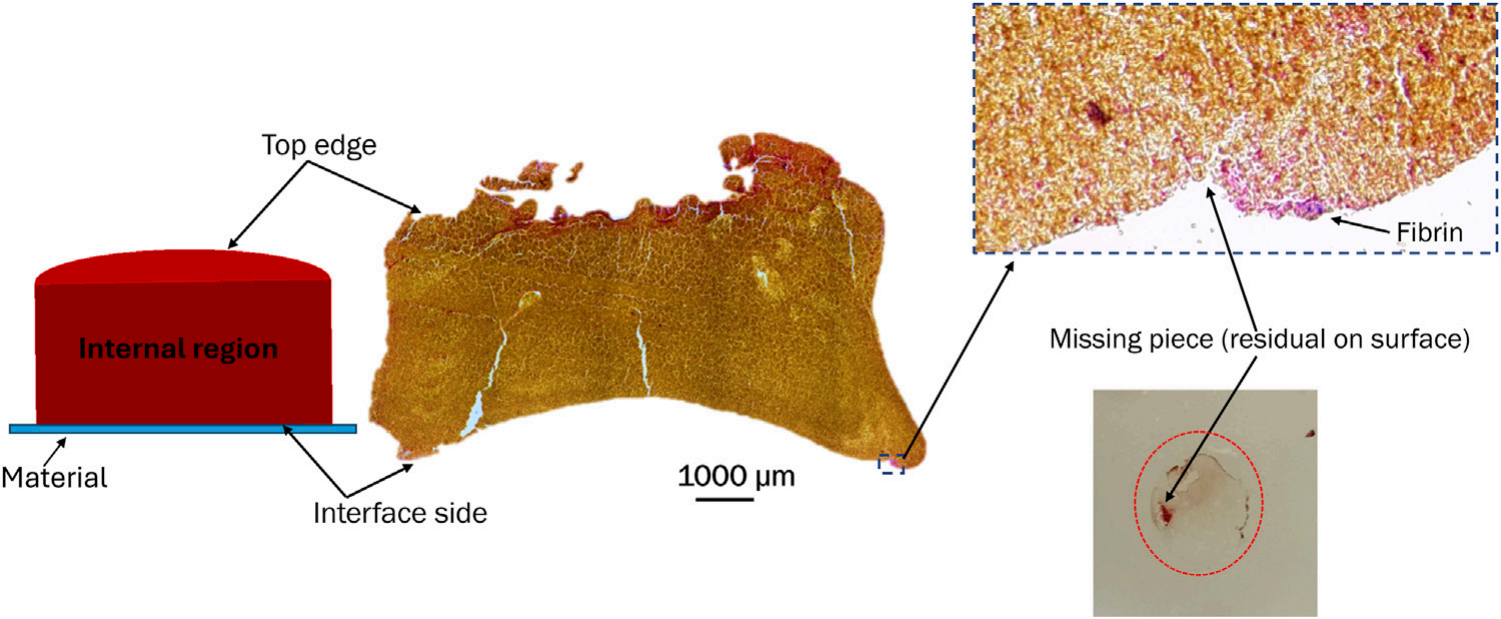
Representative image of a clot incubated for 3 h on a polyurethane surface. The image shows the distribution of fibrin, platelets, and red blood cells on the top, internal region, and interface side of the clot. Color scheme: blue (platelets), red/pink (fibrin), and yellow (red blood cells).

A histological analysis was performed to examine the clot’s structural characteristics at different incubation times. Figure 11 shows a representative image of the clot structure incubated for 3 h on polyurethane. A higher fibrin concentration occurred on a small section of the interface side (clot-material interface) of the clot. This specific region aligned with the clot’s residual portion on the surface.

**FIGURE 12:**
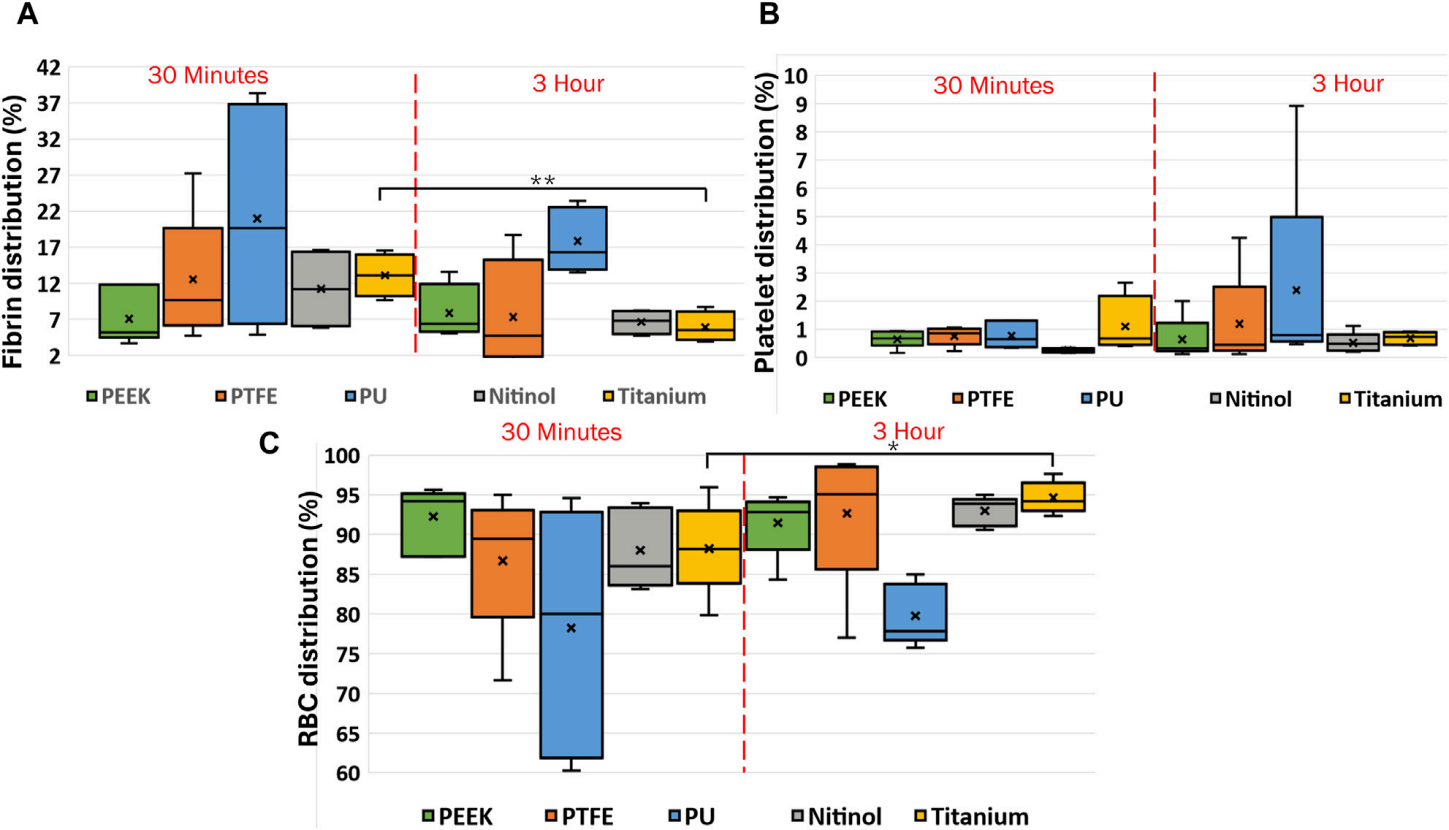
Box charts showing the percentage of **(A)** fibrin, **(B)** platelets, and **(C)** RBCs in clots incubated on different surfaces at different incubation times. After testing, the clots were fixed in 4% PFA for the histology analysis following the Carstairs protocol and imaged using a fluorescent microscope (Olympus, Tokyo, Japan. The figure shows the distribution of clot components obtained after processing the raw images using a custom MATLAB script. Asterisks denote statistical significance (*p* < 0.05 and *p* < 0.01 = **).

The clot composition was analyzed by quantifying fibrin, red blood cells, and platelets in terms of the percentage area covered in the image. Images of clot’s cross-section (Figure 2B) were considered for quantification purposes. The distribution of clot components at different incubation times is shown in Figure 12. Statistically, a significant difference was observed for clots formed on titanium surfaces. The percentage changes in fibrin, platelets, and red blood cells in clots on the titanium surface from 30 min to 3 h of incubation were as follows: −44.31%, −42.31% (statistically, insignificant), and 9.52%, respectively. The quantified mean values of clot composition are provided in (Supplementary Table S1).

**FIGURE 13:**
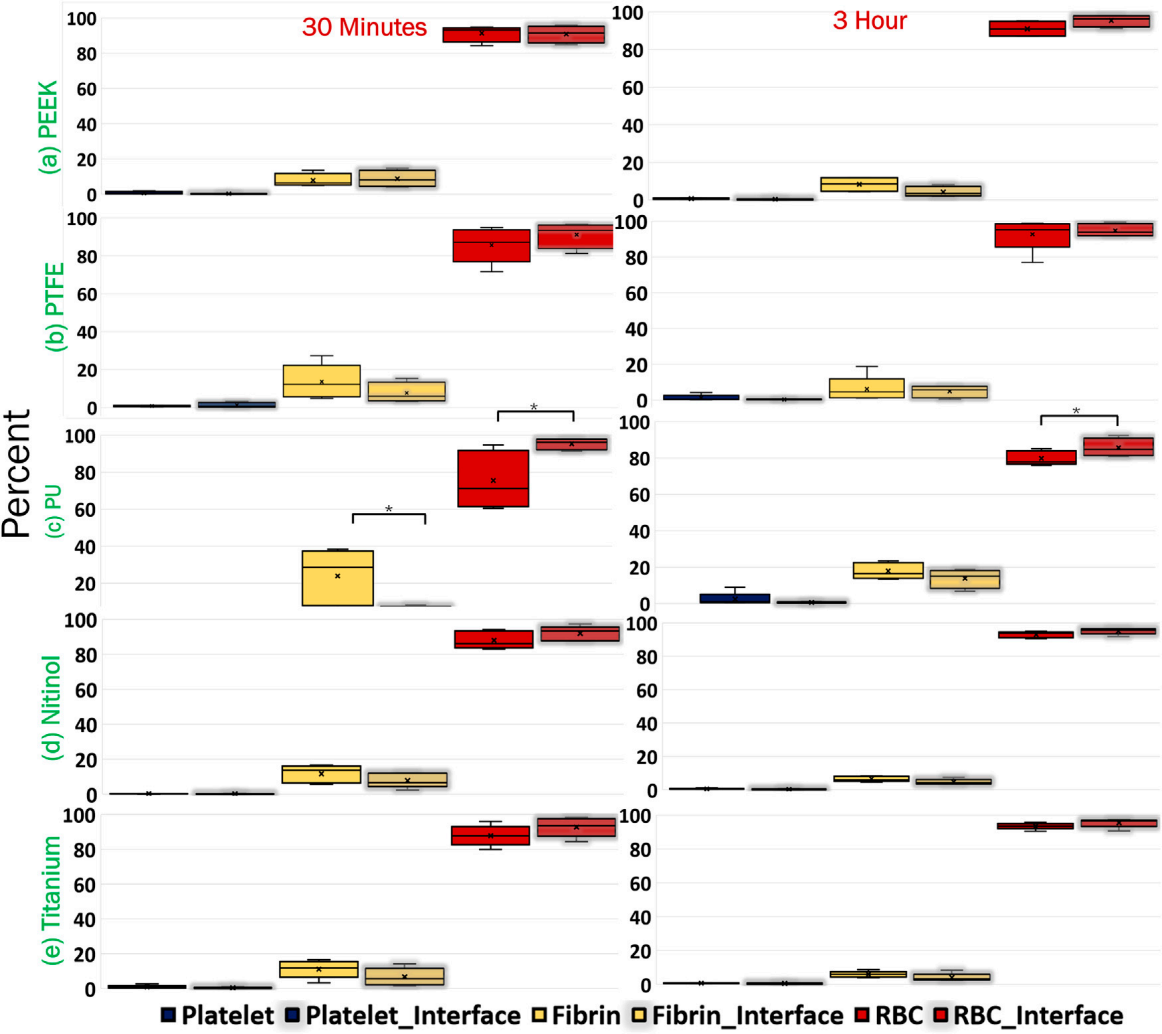
Comparison of clot composition at the cross-section and the interface (clot-material interface) for different materials. The clot samples were imaged after Carstairs staining at the cross-section and clot-material interface (representing the attachment side) and quantified to determine the percentage composition of fibrin, platelets, and red blood cells (RBCs). The interface attachment side is represented by a shaded region on the graph. Asterisks denote statistical significance (*p* < 0.05).

To analyze the change in clot composition at the interface, images at the interface were quantified to obtain the percentage of fibrin, platelets, and RBCs. Images showing the internal region, top, and interface side were quantified (Figure 2B). The quantification of clot structure at its interface was performed by taking an average of five smaller images covering the entire interface region. A comparison of the distribution of clot composition at the interface side and clot cross-section is presented in Figure 13 for clots formed on different surfaces. A significant difference was noticed in the fibrin and RBC distribution at the overall cross-section when compared to the interface side of clots on the polyurethane surface. This signifies that the clot composition can vary with the incubation time at the interface region. Moreover, across all materials, there was an observed increase in RBC concentration at the attachment region, which further increases over time.

### 3.4 Clot deformation

**FIGURE 14:**
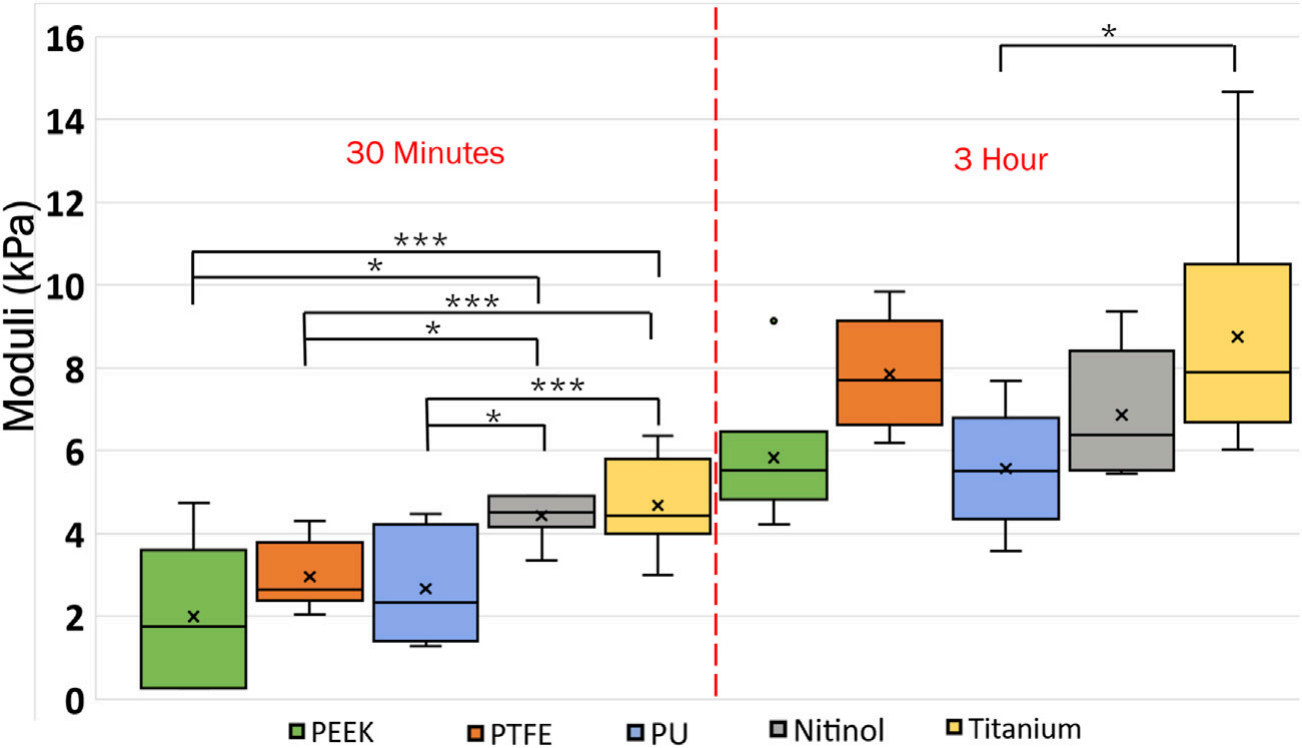
Data showing the variation of clot’s moduli with incubation time at different surfaces. The level of significance difference is represented by an asterisk (*) on the plot, where *p* < 0.05 = *, *p* < 0.01 = **, and *p* < 0.001 = ***. Symbol “•” represents the outlier.

In this study, clot deformation was characterized in terms of moduli and Poisson’s ratio. From the nominal stress-strain curve, moduli were calculated for clots on the biomaterials for the ranging incubation times (Figure 14). The data illustrate that shorter incubation periods yield lower clot moduli. Here, a low modulus value signifies higher axial stretch in the clot at the same level of stress. Further, significant changes in moduli were noticed across the materials in relation to their incubation times. For instance, at 30 min of clot incubation, titanium had the highest modulus, followed by nitinol. The mean moduli for the clots formed on the titanium surface were calculated as 5.94 ± 1.73 kPa, 33.18% higher than nitinol and at least 100.63% higher than polymers. On the other hand, clots on polymeric surfaces have lower and almost comparable moduli. A detailed insight into the impact of incubation time on the moduli is depicted in (Supplementary Figure S3).

**FIGURE 15:**
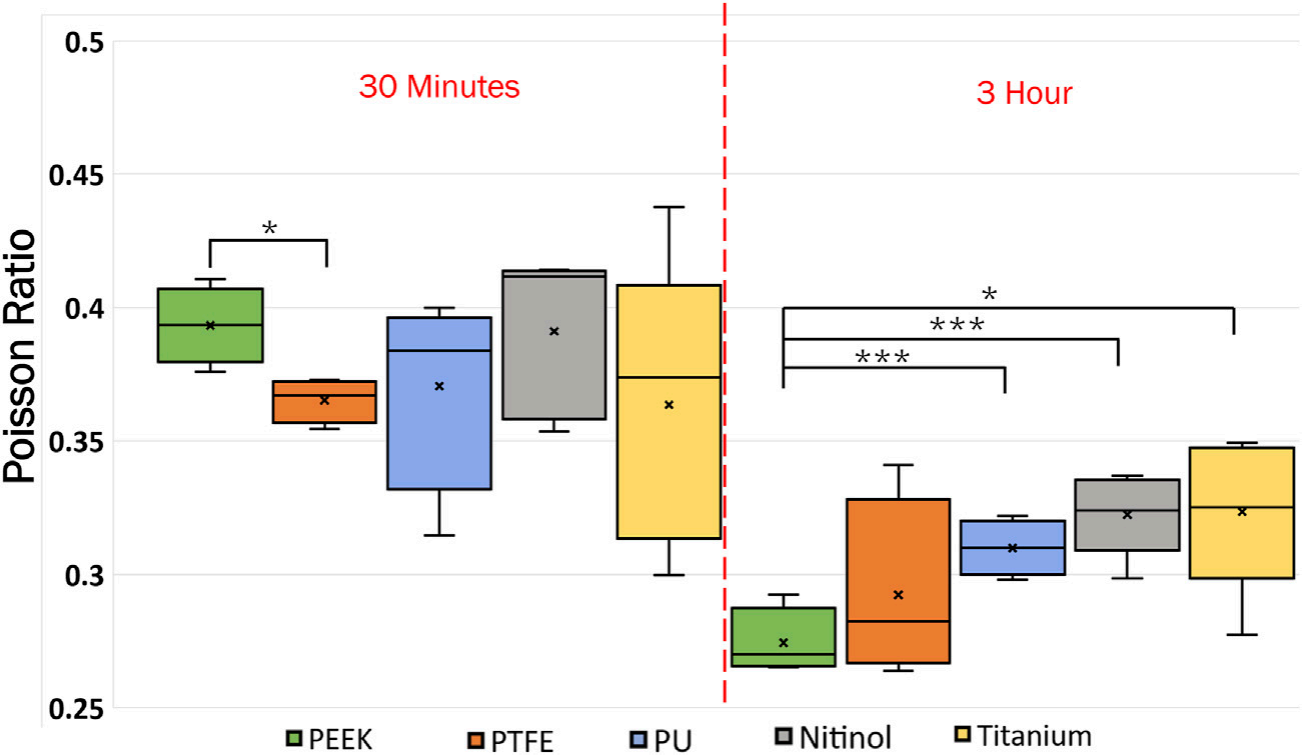
Data showing the variation of Poisson ratio with incubation time at different surfaces. The level of significance difference is represented by an asterisk (*) on the plot, where *p* < 0.05 = *, *p* < 0.01 = **, and *p* < 0.001 = ***.

Poisson’s ratio for the clots was estimated using image analysis of detachment videos taken during the experiments. The clot’s initial and final lateral and longitudinal dimensions were measured to approximate the Poisson’s ratio for clots on different surfaces. The measurement was taken by considering the attachment on the material. The variation of Poisson’s ratio with materials and incubation time is presented in Figure 15. From the data, Poisson’s ratio was inversely related to incubation time. For 3 h incubated clots, the mean Poisson’s ratio ranged between 0.3 and 0.32 for most materials. However, slightly stiffer clots are observed on PEEK surfaces (mean Poisson’s ratio: 0.28). At higher incubation, the increased cross-linking of fibrin strands and platelet-mediated contraction resulted in lower lateral movement in clots. The clots became more resistant to deformation in both lateral and longitudinal directions. The variability of the Poisson ratio with incubation time is shown in Supplementary Figure S4 in the supplementary document.

## 4 Discussion

### 4.1 Clot adhesion on biomaterials

For the first time, the adhesive characteristics of whole blood clots on different materials (PEEK, PTFE, PU, nitinol, titanium) were assessed. The clot adhesion strength showed a reliance on the material type, exhibiting variability across different materials due to differences in blood-material interaction, similar to Hanson et al., 2020 (Hanson et al., 2020). Further, a temporal variation of adhesion was observed whereby the adhesion strength increased with the incubation time. The higher percentage rise in nominal detachment stress on polymeric surfaces indicated that the temporal influence was more profound on polymers than on metals. However, the highest adhesion strength was observed on titanium surfaces, surpassing other materials by at least 35%. Stronger adhesion on titanium indicates lower embolization potential than the other materials tested but remains possible. The variation in clot adhesion on polymers and metals can be due to various material properties, including surface energy, biological response, texture, and roughness (Sarkar et al., 2007; Bullock and Bussy, 2019; Manivasagam et al., 2021; Alipour et al., 2022). The titanium samples used in this study had comparatively higher surface roughness (Sq = 0.51 ± 0.13 mm, Table 1) than other materials. A rough surface can promote higher platelet adhesion (Linneweber et al., 2007) than a smooth surface and may result in higher adhesion. In this regard, the quantification of the clot’s adhesion strength on different materials can help to improve the understanding of surface modification strategies for cardiovascular devices like coatings, polishing, texturing, *etc.*, particularly in applications where minimizing clot embolization is desirable (Parada et al., 2020). The adhesion strength of a clot to a biomaterial *in vivo* also depends on dynamic conditions like variable flow rates, which were explored previously by our group (Tobin et al., 2023).

### 4.2 Temporal dynamics of clot and its interaction with biomaterials

Clot deformation characterization revealed a direct relationship between the clot’s moduli and incubation time, as shown in Figure 14, where moduli were observed to increase with incubation time. This relationship indicated the temporal nature of the clot’s elastic behavior. As the clot matured with the incubation time, it underwent various biochemical and biomechanical events that influenced its mechanical properties. Initially, the clot’s structure is characterized by a loosely connected fibrin network with a relatively low modulus and higher Poisson’s ratio (Chandrashekar et al., 2018; Dwivedi et al., 2021; Tutwiler et al., 2021). As the clot matures, the initial fibrin matrix undergoes cross-linking yielding a denser and stiffer network that enhances the clot’s resistance to deformation. Simultaneously, with time platelet contraction within the fibrin network reduces the pore size and compacts the structure (Supplementary Figure S5), further increasing the modulus (Pathare et al., 2021). Similar changes in the clot’s mechanical properties have been reported under shear flow conditions, where the clots exhibit an increase in stiffness as they mature (Chen et al., 2019). The shear forces facilitate fibrin network alignment and platelet integration, resulting in a more mechanically stiffer clot with time (Chen et al., 2019). These findings are consistent with our observations in a biomaterials system, where similar maturation effects were noted, although in a static environment.

Clots incubated for longer times were stiffer prior to detachment, leading to a higher detachment stress (see Figure 4). In a clinical context, such clots are less likely to break apart, reducing the risk of distal embolization. Incubation time is particularly crucial in thrombectomy procedures. The general therapeutic window for stroke interventions is approximately 6 h (Saver et al., 2016). Longer incubation periods may lead to less favorable outcomes (Saver et al., 2016) due to the changes in thrombus composition over time (Abbasi et al., 2022). Notable differences in clot’s structure in terms of percentage change in fibrin and red blood cells are also observed on titanium in this study as the incubation time varied from 30 min to 3 h (see Figure 12; Supplementary Table S1). For instance, at 30 min incubation, clots adhered to titanium showed the highest modulus, with mean moduli calculated as 7.32 ± 1.73 kPa (remarkably 73% higher than other materials, Figure 14), followed by nitinol. This observation emphasizes the time dependent influence of clot-material interaction on the clot’s mechanical properties and suggests lower deformability for clot adhered to titanium surfaces (Figures 14, 15). The quantitative analysis of clot remnants post-detachment revealed a larger remnant region with longer incubation time on all surfaces, as shown in Figure 5. A larger remnant area signifies a comparatively higher adhesive region on the surface, resisting the detachment. This trend is more evident on metallic surfaces than polymers. These potential embolization sites with remnants may serve as locations for clot regrowth and increase stroke and myocardial infarction risk, where it may be possible that subsequent growth could be less adhesive with higher embolization risk. Further, confocal microscopy analysis revealed temporal variations in the fibrin-platelet network distribution of the clot remnant across the biomaterial surfaces (see Figures 8–10). Higher fibrin-platelet density was noted at longer incubation times, which was consistent with platelet distribution over time (Takahashi et al., 2019). Imaging on the remnant sites showed a well-connected platelet-fibrin region that yields a stronger clot-surface attachment than the remnant-free areas. Among the polymers, PTFE exhibited the highest adhesion strength, possibly due to more significant, well-connected platelet-fibrin regions on its surface (see Supplementary Figure S6; Supplementary Material). This highlights the time-dependent nature of clot’s adhesive behavior, which is further influenced by the choice of material substrate.

### 4.3 Importance of material-dependent coagulation in cardiovascular devices

The current study demonstrated that a clot adheres non-homogenously to a material surface due to the non-uniform distribution of platelets and fibrin over the surface. Furthermore, the clot adhesion increases with time due to a well-connected platelet-fibrin mesh that is influenced by material dependent, initial coagulation. The array of materials used in this study generally have excellent biocompatibility but still have thrombogenic potential. For instance, rapid platelet adhesion has been reported on rough titanium surfaces (Nakamura et al., 2020), while significant platelet adhesion has also been observed on untreated nitinol surfaces (Gegenschatz-Schmid et al., 2022). Among polymers, PTFE is generally a smooth hydrophobic surface that helps minimize protein adsorption and platelet adhesion (Liu et al., 2022). Whereas PEEK showed thrombogenic potential due to higher platelet adhesion in both bench testing and a short-term animal model (Tateishi et al., 2014; Kambe et al., 2019). A moderate thrombogenicity potential was also reported for polyurethane (Medical Device Material Performance Study, 2024). This showed that material surfaces, whether treated or untreated, possess an inherent thrombogenic potential that varies among materials. This thrombogenic tendency of the material plays a significant role in influencing the clot adhesive characteristics.

The histological analysis revealed differences in the distribution of fibrin, red blood cells, and platelets between the clot’s interface and its cross-section. For all materials, the histological data revealed a higher RBC concentration at the clot’s interface region than its cross-section (Figure 13). Furthermore, for polyurethane, a significant difference in the distribution of fibrin and RBC was noticed. Whether the time scales for attachment and coagulation are on the same order is not yet fully understood, yet it is well known that coagulation is highly dependent on the substrate material properties, as shown by Xu et al., 2014 (Xu et al., 2014), and we now show that this material-dependent coagulation drives attachment strength.

This study presents the first quantification of the time-dependent, adhesive behavior of blood clots on medical device-related biomaterials. In the context of clot mechanical characterization, previous studies have focused on the internal fracture of blood clots, rather than a detachment/embolization stress. Tutwiler et al., 2020 (Tutwiler et al., 2020) reported uniaxial fracture propagation stresses of roughly 2 kPa without a defect present, and 1.3 kPa with a relatively large defect present (notched sample). Sugerman et al., 2020 (Sugerman et al., 2020) demonstrated clot deformations up to 40% strain, with peak stresses of 3 kPa for bovine clots, prior to fracture. Our findings indicate that aggregate clot detachment occurs from 0.3 kPa to 1.06 kPa overall within 3 h of incubation time, correlating to clot fracture with a prescribed tear (‘notch’).

Statically formed clots, as used in this study, may not replicate the dynamic blood condition that occurs *in vivo*. In comparison to a static environment, clot formation in a dynamic system can also be influenced by shear forces and the continuous replenishment of blood factors. *In vivo*, the dynamic environment contributes to the layered structure of clots, where fibrin and platelets are distributed differently compared to static clots (Xu et al., 2012). The replenishment of clotting factors and fibrin may result in different mechanical properties of the clot than the static system (Link et al., 2020). Although clot formation in static and dynamic environments may differ, the adhesion characteristics of clots may remain relatively consistent. For instance, in a dynamic flow condition, a clot adhered to an implantable medical device or a blood vessel, the clot-material interface may behave similarly to an area of flow stasis. Further, previous studies showed similarities in platelet aggregate formation under laminar flow and static conditions (O'Brien, 1990; Jackson et al., 2009; Nesbitt et al., 2009). However, the adhesion strength may vary due to the presence of shear flow conditions. In this regard, the detachment stress calculated in this study was of the same order as the shear detachment stress reported by Tobin *et al.* for flow -induced embolization (Tobin et al., 2023), suggesting that clot embolization from a surface behaves similarly to a uniaxial detachment. Further, the detachment forces observed in this study were comparable to the thrombectomy forces reported by Romero et al., 2010 (Romero et al., 2010) and Shi et al., 2017 (Shi et al., 2017). This observation indicated potential similarities in the mechanisms of clot interfacial failure demonstrated in this study and those associated with flow and shear-induced embolization (Romero et al., 2010; Shi et al., 2017; Tobin et al., 2023). Currently, there is a lack of studies specifically investigating detachment stress in similar experimental settings for further validation. Translating the findings of this study to a real-world physiological dynamic system will require additional studies.

The results of the current study suggest a higher variation of clot composition on the titanium surface than on other materials. The higher variability of clot structure may be due to the early cell recruitment characteristics of the titanium surface (Yang et al., 2016). This variability can be attributed to the surface properties influencing the adhesion and activation of blood cells, leading to differences in clot formation and organization (Jaffer et al., 2015a). This highlights the importance of carefully considering materials for cardiovascular devices, as the risk of embolization depends on the material used. The material-dependent coagulation, which simultaneously influences clot internal and interfacial properties can be beneficial in understanding and improving blood-contacting surfaces in future studies to ensure the optimal performance and longevity of implantable cardiovascular devices. The findings of this work are also relevant to clinical therapies such as dual antiplatelet therapy (DAPT) and anticoagulant therapies aimed at reducing thrombus formation in implantable medical devices and vascular surfaces. Despite DAPT and anticoagulant therapies, device thrombus persists (Plicht et al., 2013; den Exter et al., 2020), emphasizing the critical role of material selection in minimizing embolization risk. This study provides insights into how surface properties influence clot adhesion characteristics. Future studies should explore the specific impacts of anticoagulation on clot adhesion using the methodology demonstrated in this study, anticipating variations in adhesion strength under these conditions.

### 4.4 Limitations

The blood clots used in this study were formed statically. The properties of statically formed clots can differ from those of dynamically formed clots (Preut et al., 2018). Furthermore, the incubation time in this study was limited to 3 h as opposed to the 6-h stroke therapeutic window (Saver et al., 2016). Platelet-rich clots can also form on implanted cardiovascular devices (Jessen et al., 2021), but these types of clots were not tested in this study. In this work, bare materials were used without additional surface treatments, such as polishing or smoothing. Surface roughness is recognized as a critical parameter in determining adhesion strength, and standardizing roughness levels with appropriate surface treatment could influence the clot-material interaction outcomes observed in this study. This is a first approximation study, primarily focused on developing a new experimental method and examining the effects of incubation time and general material differences. Future research will address this limitation by incorporating surface treatments to equalize roughness levels across different materials and investigating the effects of varying roughness levels. This study serves as a foundational step, with plans to build on these findings by systematically exploring the role of surface treatments and roughness in subsequent studies. Nevertheless, this work provides an understanding of the adhesion of blood clots on different biomaterials. This work has shown that clot adhesive behavior exists, which has not been explored in previous studies. The temporal adhesive characteristics of clots can influence device-induced embolization, which may also impact the efficacy of clot extraction in embolectomy surgeries.

## 5 Summary

This study investigated clot adhesion on cardiovascular device materials, such as PTFE, PU, PEEK, nitinol, and titanium. Clot detachment demonstrated that clots on titanium surfaces exhibited the highest adhesion strength. Clot adhesion varied across materials, with polymers showing a more pronounced time-dependent behavior. Post-detachment analysis revealed non-uniform cellular distribution on surfaces, with metallic surfaces having larger remnants, indicating stronger adhesion regions. Confocal analysis revealed differences in platelet-fibrin network intensity across materials and incubation times. Further, changes in clot structure were also noticed amongst the cross-section and interface side of the clot. Incubation time can also impact clot deformability, which further varies with materials. Overall, this study demonstrated temporal variations in clot adhesion and material-dependent clot mechanical properties. These findings could inform strategies to enhance mechanical thrombectomy outcomes by reducing embolic complications caused by blood-contacting cardiovascular devices.

## Data Availability

The raw data supporting the conclusions of this article will be made available by the authors, without undue reservation.
