# Supplementary material for: Characterizing thrombus adhesion strength on common cardiovascular device materials
Source: Front Bioeng Biotechnol. 2024 Aug 14;12:1438359. doi: 10.3389/fbioe.2024.1438359 (PMC11349534; doi:10.3389/fbioe.2024.1438359)
Supplement: Supplementary file 1 [file DataSheet1.docx]

**Supplementary Materials**

**Table S1:** Distribution of fibrin, platelet, and RBCs for clots on different surfaces at 30 minutes and 3-hour incubation. Asterisks denote statistical significance between 30 minutes and 3-hour clots (p < 0.05).

| **Fibrin** | | | | | |
| --- | --- | --- | --- | --- | --- |
|  | **PEEK** | **PTFE** | **PU** | **Nitinol** | **Titanium*** |
| **30 Minutes** | 7.07 ± 3.25 | 12.54 ± 7.76 | 20.98 ± 13.89 | 11.74 ± 4.51 | 10.23 ± 4.47 |
| **3 Hour** | 7.87 ± 3.39 | 6.10 ± 6.77 | 17.84 ± 3.97 | 6.47 ± 1.49 | 5.94 ± 1.82 |
| **Platelets** | | | | | |
|  | **PEEK** | **PTFE** | **PU** | **Nitinol** | **Titanium** |
| **30 Minutes** | 0.65 ± 0.26 | 0.76 ± 0.29 | 0.78 ± 0.40 | 0.26 ± 0.06 | 0.82 ± 0.81 |
| **3 Hour** | 0.64 ± 0.68 | 1.20 ± 1.53 | 2.39 ± 3.27 | 0.61 ± 0.30 | 0.74 ± 0.20 |
| **Red Blood Cells** | | | | | |
|  | **PEEK** | **PTFE** | **PU** | **Nitinol** | **Titanium*** |
| **30 Minutes** | 92.27 ± 3.58 | 86.71 ± 7.89 | 78.25 ± 14.18 | 88.06 ± 4.50 | 88.62 ± 5.20 |
| **3 Hour** | 91.77 ± 4.08 | 92.69 ± 8.42 | 79.75 ± 3.44 | 92.99 ± 1.75 | 94.24 ± 2.49 |


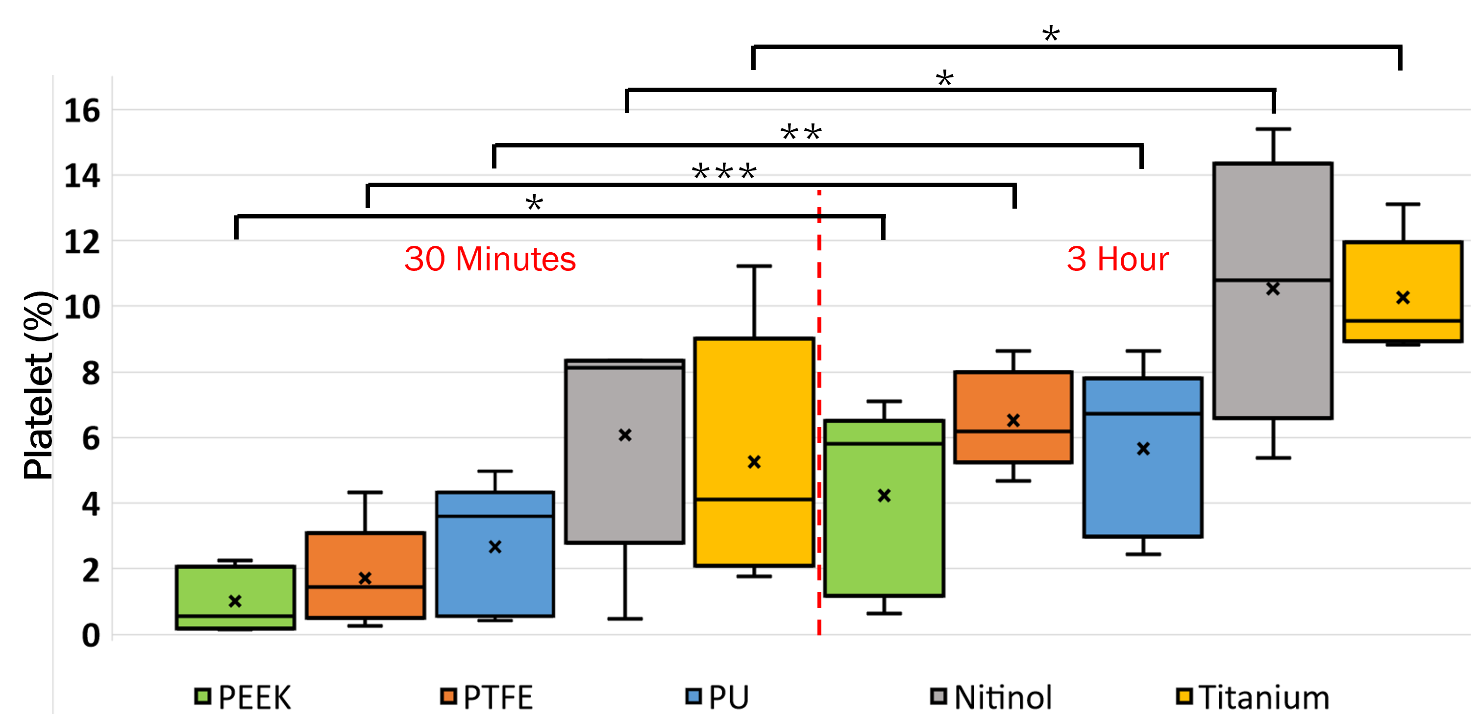


Figure S1: Quantification of platelets coverage on the surface of biocompatible materials at the remnant regions at different incubation times. Asterisks denote statistical significance between two-time intervals (p < 0.05 = *, p < 0.01 = **, and p < 0.001 = ***).


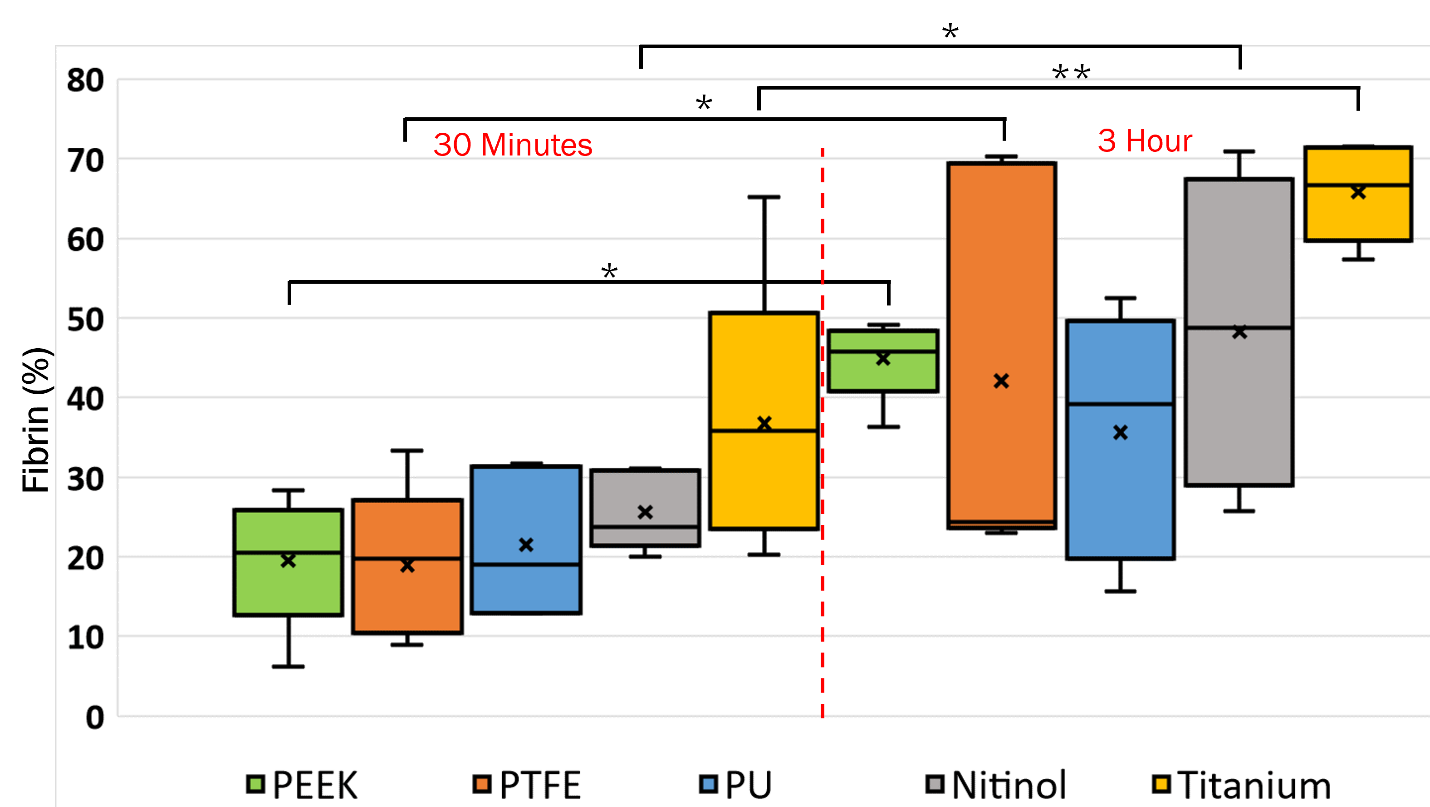


Figure S2: Quantification of fibrin coverage on the surface of biocompatible materials at the remnant regions at different incubation times. Asterisks denote statistical significance between two-time intervals (p < 0.05 = *, and p < 0.01 = **).


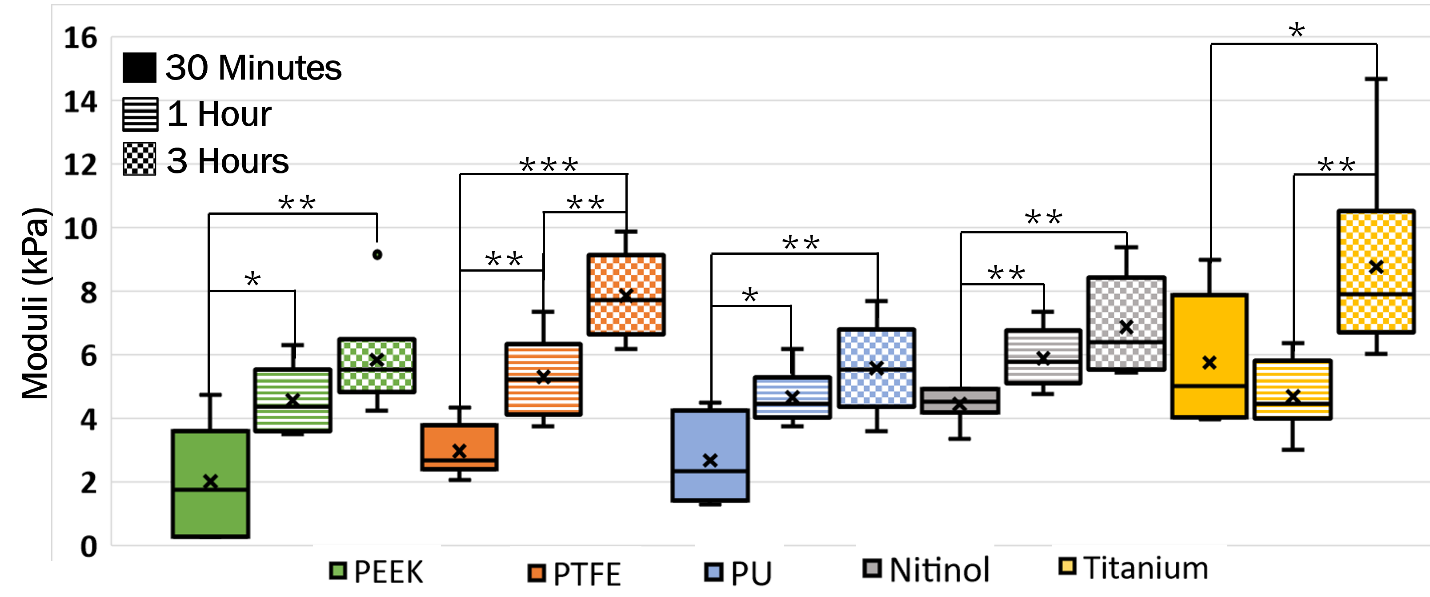


Figure S3: Data depicting the influence of incubation time on clot moduli for different surfaces. Significance levels are denoted on the plot by asterisks (*), indicating p-values where p < 0.05 = *, p < 0.01 = **, and p < 0.001 = ***. The symbol '•' denotes outliers.


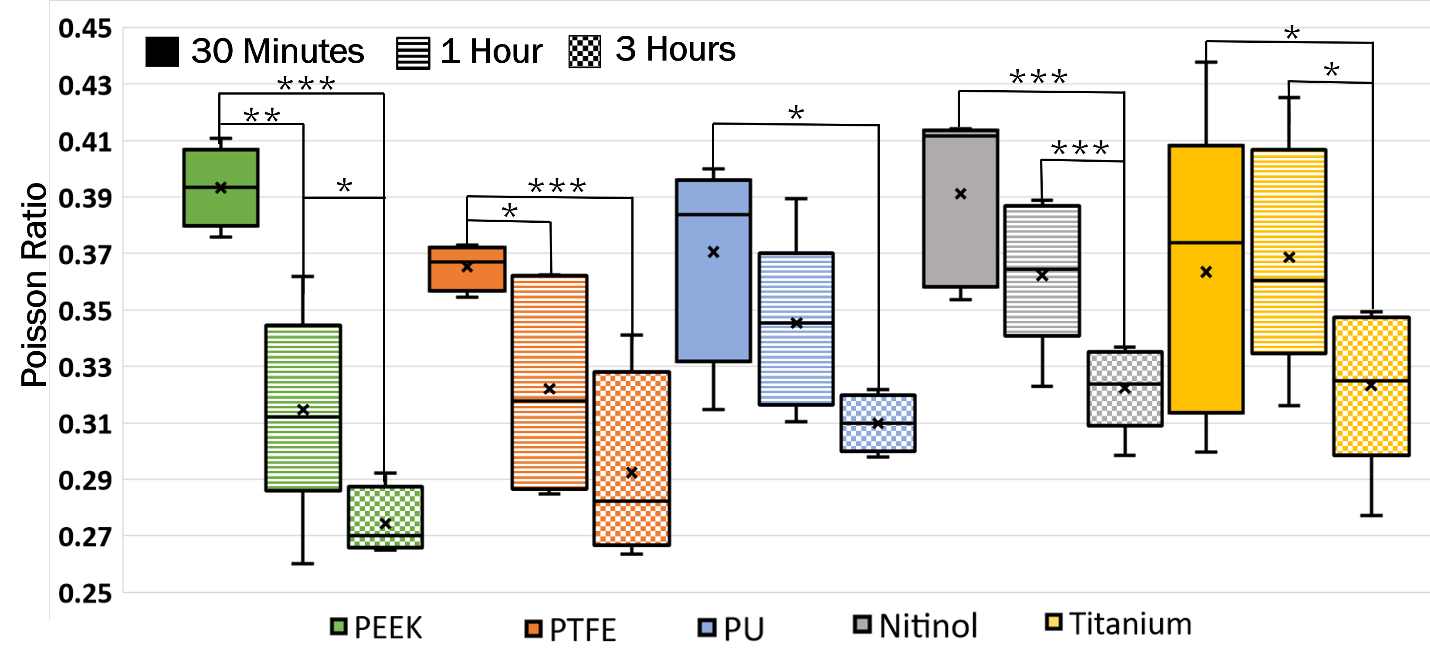


Figure S4: Data illustrating the influence of incubation time on Poisson ratio across different surfaces. Significance levels are indicated on the plot using asterisks (*), with p-values represented as p < 0.05 = *, p < 0.01 = **, and p < 0.001 = ***.


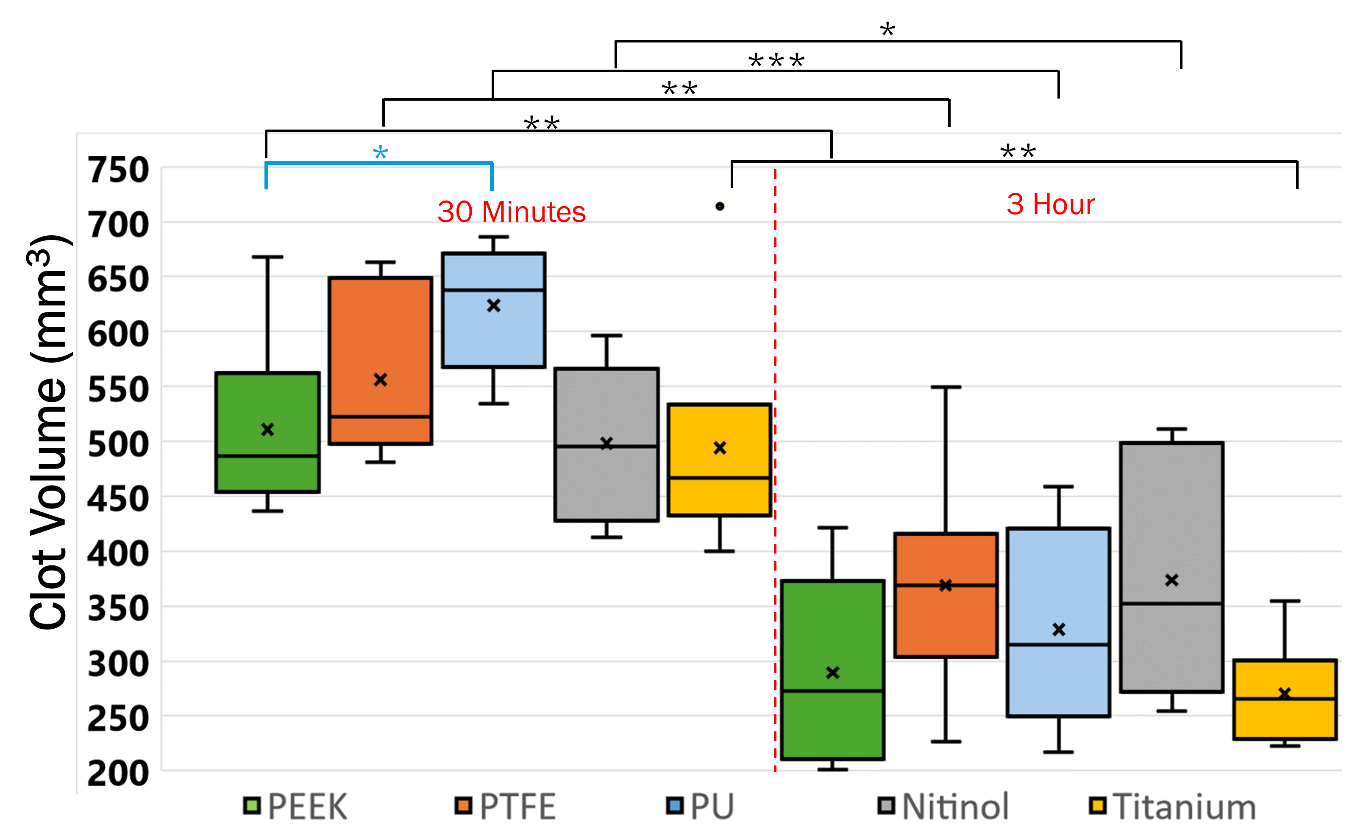


Figure S5: Data showing variations in clot volume over incubation time. Significant changes in clot volume were observed with increasing incubation periods. Furthermore, significant differences were identified among clots formed on PEEK and PU at 30 minutes of incubation (shown by blue lines on the plot), indicating distinct coagulation behavior of blood on different surfaces. Significance levels are indicated using asterisks (*): p < 0.05 = *, p < 0.01 = **, and p < 0.001 = ***. Symbol “•” represents the outlier.


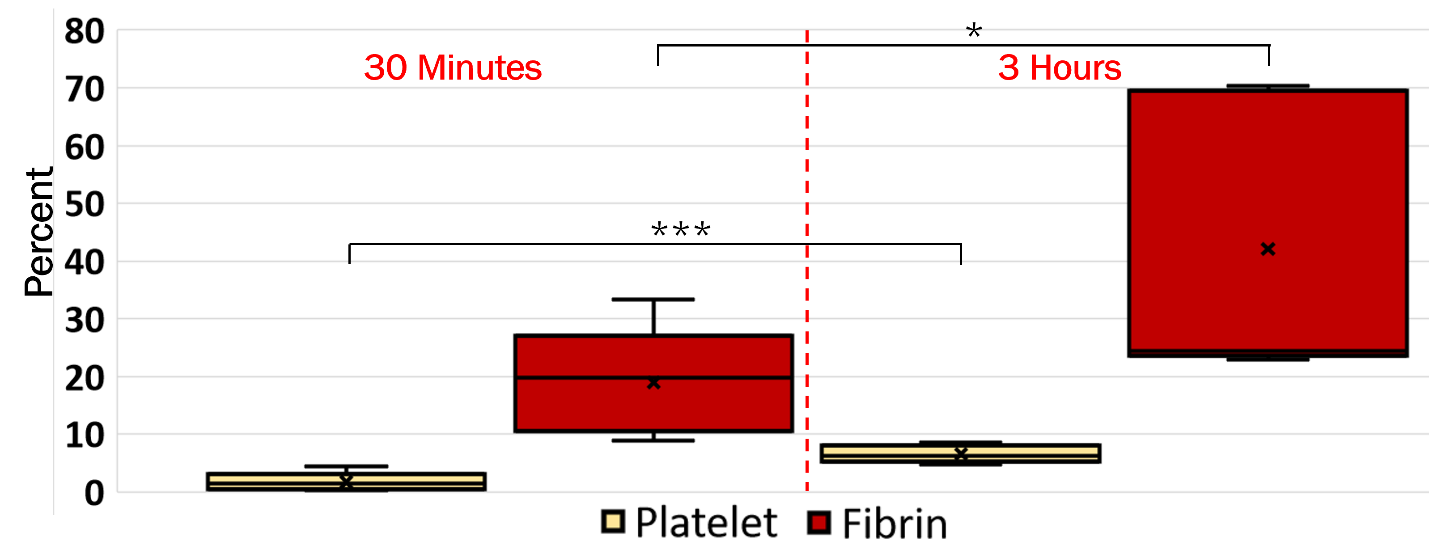


Figure S6: Quantification of platelet and fibrin coverage on the PTFE remnant regions at different incubation times. Asterisks denote statistical significance between two-time intervals (p < 0.05 = *, and p < 0.001 = ***).
